# Dysentery

**Published:** 1850-05

**Authors:** 


Dysentery.—Extract from a letter to the editor. "I must say, yet with all due deference to the opinion of others, that I believe all purgatives of any kind, out of place in the treatment of dysentery. The disease I believe primarily to consist in functional derangement of the liver, and inflammation of the mucous membrane of the colon and rectum; other portions of the bowels, however, may also be involved in the disease. I have had no satisfactory opportunity of testing the plan of treatment, based upon the above pathological conditions, until last autumn, when the disease prevailed epidemically in this region. It did not appear to have any peculiarities not common to dysentery generally, save perhaps greater tendency to a typhoid condition than usual. I commenced the treatment by giving full doses of opium, in combination with small portions of Ipecacuanha, repeated at such intervals as to keep the pain and tenesmus in subjection; blue pill was also given in three or four grain doses, at intervals of four hours, and continued until slight symptoms of mercurialization obtained, unless the disease gave way before. Active counter-irritation was kept up, with strong volatile liniment throughout; a milk diet, and mucilaginous drinks, were also rigidly enjoined. Out of one hundred and two cases, treated on the above plan, ninety-eight recovered in from two to six days; the four fatal ones, were all in an advanced state, when they were brought under treatment. J. HOHE.

*Ohio Med. and Surg. Journal.*

